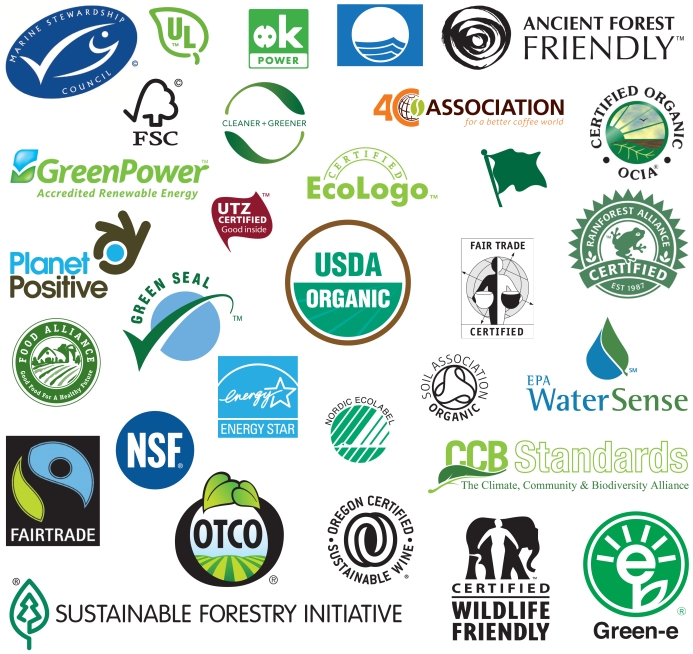# Green Washing: Do You Know What You’re Buying?

**DOI:** 10.1289/ehp.118-a246

**Published:** 2010-06

**Authors:** Richard Dahl

**Affiliations:** Boston freelance writer **Richard Dahl** has contributed to *EHP* since 1995. He also writes periodically for the Massachusetts Institute of Technology

In a United States where climate change legislation, concerns about foreign oil dependence, and mandatory curbside recycling are becoming the “new normal,” companies across a variety of sectors are seeing the benefit of promoting their “greenness” in advertisements. Many lay vague and dubious claims to environmental stewardship. Others are more specific but still raise questions about what their claims really mean. The term for ads and labels that promise more environmental benefit than they deliver is “greenwashing.” Today, some critics are asking whether the impact of greenwashing can go beyond a breach of marketing ethics—can greenwashing actually harm health?

## Greenwash: Growing (Almost) Unchecked

Greenwashing is not a recent phenomenon; since the mid-1980s the term has gained broad recognition and acceptance to describe the practice of making unwarranted or overblown claims of sustainability or environmental friendliness in an attempt to gain market share.

Although greenwashing has been around for many years, its use has escalated sharply in recent years as companies have strived to meet escalating consumer demand for greener products and services, according to advertising consultancy TerraChoice Environmental Marketing. Last year TerraChoice issued its second report^2^ on the subject, identifying 2,219 products making green claims—an increase of 79% over the company’s first report two years earlier.^3^

TerraChoice also concluded that 98% of those products were guilty of greenwashing. Furthermore, according to TerraChoice vice president Scot Case, the problem is escalating. TerraChoice also measured green advertising in major magazines and found that between 2006 and 2009, the number mushroomed from about 3.5% of all ads to just over 10%; today, Case says, the number is probably higher still. Case says researchers are currently working on another update that will be released later this year, and he predicts the number of products making dubious green claims will double.

Compounding the problem is the fact that environmental advertising—in the United States, at least—is not tightly regulated. The Federal Trade Commission (FTC), the agency responsible for protecting the public from unsubstantiated or unscrupulous advertising, does have a set of environmental marketing guidelines known as the Green Guides. Published under Title 16 of the *Code of Federal Regulations*,^4^ the Green Guides were created in 1992 and most recently updated in 1998. According to Laura DeMartino, assistant director of the FTC Division of Enforcement, the proliferation of green claims in the marketplace includes claims that are not currently addressed in the Green Guides, and updated guidance currently is being developed.

The FTC originally planned to begin a review of the Green Guides in 2009, but the commission moved the schedule up, according to DeMartino, in response to a changing landscape in environmental marketing. “The reason, at least anecdotally, was an increase in environmental marketing claims in many different sectors of the economy and newer claims that were not common, and therefore not addressed, in the existing Guides,” she says. “These are things like carbon offsets or carbon-neutrality claims, terms like ‘sustainable’ or ‘made with renewable materials.’”

The FTC held a series of workshops in 2008, holding separate events for each of three areas: carbon-offset and renewable-energy claims, green packaging, and buildings and textiles. In association with each workshop, the FTC asked for comments to help shed light on consumer perception of green advertising, but DeMartino says the commission received very few. The FTC responded to this gap by commissioning a research firm, Harris Interactive, to provide that information. DeMartino says that research has been completed, and a report on it will accompany the revision announcement, which is expected soon.

## How Updated Guidance Might Look

In aspiring to revise its environmental marketing guidelines, the FTC is following a trend that has been evident in other nations. In 2008, the Canadian Competition Bureau (a government agency similar in function to the FTC) updated its environmental marketing guidelines to reduce green misinformation,^5^ and the Australian Competition and Consumer Commission took a similar step.^6^ In March 2010, the U.K. Committee of Advertising Practice and Broadcast Committee of Advertising Practice announced an update to their codes of practice designed to curtail greenwashing.^7^

All three updates are “remarkably similar,” Case says, but he suggests the Canadian revisions might provide the best “sneak peak” at what the FTC might do because the two agencies have a long history of working together on cross-border consumer matters. Attorney Randi W. Singer, a litigation partner at New York’s Weil Gotschal who has defended companies accused of false advertising, agrees the moves made in Canada but also in Australia and the United Kingdom may provide a good look at what is to come in the United States. Those changes, coupled with her own analysis of the FTC workshop discussions, provide the basis for her to make several predictions about what the new U.S. regulatory scheme might look like.

Singer predicts the revisions will probably contain new definitional language for terms such as “carbon neutral” and “sustainable.” She also expects the FTC will address the issue of third-party certifications—that is, the plethora of green labels consumers see on their products. She says the workshop discussions included “a lot of talk about the need for standardization of certifications, a need to have a process for certifications so it’s not just people registering themselves, a need to standardize the iconography and the testing.”

According to Case, there are now more than 500 green labels in the United States, and some are “significantly more meaningful” than others. “I testified before Congress last summer^8^ and I pointed out a certain lawyer in Florida who set up a website and is ‘certifying’ products. He doesn’t need to see the product, he doesn’t need test results. He just needs to see your credit card number,” Case says.

Meanwhile, the FTC has begun to step up enforcement regarding claims that it considers clear violations of the existing Green Guides, last year charging three companies with false and unsupportable claims that a variety of paper plates, wipes, and towels were biodegradable.^9^ “When consumers see a ‘biodegradable’ claim they think that product will degrade completely in a reasonably short period of time after it has been customarily disposed,” DeMartino says. But for about 91% of the waste in the United States, the FTC wrote in its 2009 decisions,^10^ customary disposal means disposal in a landfill, where conditions prevent even a theoretically biodegradable item from degrading quickly.

In another instance, the FTC charged four sellers of clothing and other textiles with deceptively advertising and labeling various textile items as biodegradable bamboo that had been grown in a more sustainable fashion than conventional cotton, when, in fact, the items were rayon, a heavily processed fiber.^11^ In January 2010, the FTC sent letters to 78 additional sellers of clothing and textiles warning them they may be breaking the law by advertising and labeling textile products as bamboo.^12^

## The Health Impact of Greenwash

One major result of greenwashing, say Case and others, is public confusion. But can greenwashing also pose a threat to the environment and even to public health? Critics say greenwashing is indeed harmful, and they cite examples.

In 2008, the Malaysia Palm Oil Council produced a TV commercial touting itself in very general terms as eco-friendly; a voice-over stated “Malaysia Palm Oil. Its trees give life and help our planet breathe, and give home to hundreds of species of flora and fauna. Malaysia Palm Oil. A gift from nature, a gift for life.” But according to Friends of the Earth and other critics of the ad, palm oil plantations are linked to rainforest species extinction, habitat loss, pollution from burning to clear the land, destruction of flood buffer zones along rivers, and other adverse effects. The U.K. Advertising Standards Authority agreed, declaring the ad in violation of its advertising standards; contrary to the message of the ad, the authority ruled, “there was not a consensus that there was a net benefit to the environment from Malaysia’s palm oil plantations.”^13^

In 2008, the authority rebuked Dutch energy giant Shell for misleading the public about the environmental effects of its oil sands development project in Canada in the course of advertising its efforts to “secure a profitable and sustainable future.”^14^ While acknowledging the term “sustainable” is “used and understood in a variety of ways by governmental and non-governmental organisations, researchers, public and corporate bodies and members of the public,” the authority also noted that Shell provided no evidence backing up the “sustainability” of the oil sands project,^14^ which has been criticized widely for its environmental impact.^15^

Case contends that makers of indoor cleaning products are among the worst greenwash offenders. “People are attempting to buy cleaning chemicals that have reduced environmental and health impacts, but [manufacturers] are using greenwashing to either confuse or mislead them,” he says. “People aren’t really well-equipped to navigate the eco-babble, and so they end up buying products that don’t have the environmental or human-health performances that they expect.”

TerraChoice’s 2009 report concluded that of 397 cleaners and paper cleaning products assessed, only 3 made no unsubstantiated or unverifiable green claims.^2^ The report noted that cleaners, along with cosmetics and children’s products, are particularly prone to greenwashing—a worrisome state, given that these items are “among the most common of products in most households.”^2^

While companies see consumers’ growing demands for green products as an opportunity to increase sales by making perhaps dubious environmental claims, they may also be doing so in an attempt to avoid regulation, says Bruno. In addition to the FTC’s promises to tightening up its rules on environmental advertising, broader governmental pressures increasingly place greater burdens on producers to ensure their products are environmentally sound.

“A single ad or ad campaign may be an attempt to sway a customer. But the preponderance of green image ads, many of which are not even attempting to sell a product, combined with lobbying efforts to avoid regulation, add up to a political project that I call ‘deep greenwash,’” Bruno says. “Deep greenwash is the campaign to assuage the concerns of the public, deflect blame away from polluting corporations, and promote voluntary measures over bona fide regulation.”

However, several corporate and marketing professionals warn that growing consumer cynicism about these kinds of general campaigns make them risky ventures for companies who engage in them. Keith Miller, manager of environmental initiatives and sustainability at 3M, last year addressed a seminar of The Conference Board, a business-management organization, about what his company does to avoid greenwashing allegations. Summarizing his presentation for the business blog CSR Perspective, Miller said that, based on 3M’s experiences, he encouraged companies to avoid making “broad environmental claims” and that any claims made should be specific to products and backed up by “compelling” data.^16^

Ogilvy & Mather advertising agency, recently released a handbook designed to guide managers in how to avoid green-washing charges and called upon them to adopt a policy of “radical transparency” in green advertising campaigns.^17^ Business for Social Responsibility, a consulting and research organization, has also published a handbook, *Understanding and Preventing Greenwash: A Business Guide*, which also emphasized the need for transparency as well as for bolstering any environmental claims with independent verification.^18^

## Reining In Greenwash

In the absence of a strong regulatory scheme, consumer and environmental groups have stepped into the vacuum to keep an eye on corporate use of greenwashing. Greenpeace was one of the first groups to do so, creating a separate anti-greenwash group, stopgreenwash. org, which monitors alleged greenwash ads and provides other information on identifying and combating greenwash. The University of Oregon School of Journalism and Communication and EnviroMedia Social Marketing operate greenwashingindex.com, where people may post suspected greenwash print or electronic ads and rank them on a scale of 1 to 5 (1 is “authentic,” 5 is “bogus”).

Claudette Juska, a research specialist at Greenpeace, also points to numerous anti-greenwash blogs that have emerged. The result, she says, is that “there’s been a lot of analysis of greenwashing, and the public has caught on to it. I think in general people have become skeptical of any environmental claims. They don’t know what’s valid and what isn’t, so they disregard most of them.”

Thomas P. Lyon, a business professor at the University of Michigan who has written and spoken extensively about greenwashing, agrees. He says companies are aware they may be criticized or mocked for making even valid claims, so they’re starting to grow skittish about making green claims of any kind.^19^ “That’s why companies, I think, want to see the FTC act—to give them some certainty,” he says.

David Mallen, associate director of the National Advertising Division of the Council of Better Business Bureaus, the advertising industry’s self-regulatory body, says companies are growing increasingly aware of the dangers of greenwashing. Although some of the matters his office handles are initiated by consumers, the large majority are prompted by companies disputing competitors’ claims.

“We’re definitely seeing a rise in challenges about the truth and accuracy of green marketing and environmental marketing,” he says. “It’s certainly taking up a greater percentage of the kinds of advertising cases that we look at. Because green advertising is so ubiquitous now, there’s so much greater potential for confusion, misunderstanding, and uncertainty about what messages mean and how to substantiate them.”

Typically, he says, a company will be attacked for making a broad or general claim about a product being environmentally friendly “based only on a single attribute, which might not even be a meaningful one.” But he says many other cases focus on a competitor’s use of a word such as “biodegradable” or “renewable.” He adds, “We’re also seeing these aggressive, competitive green advertisements where a company will say ‘Not only are we green, not only are we making significant efforts toward sustainability, but our competitors aren’t.’”

Lyon says he’s found the companies that are most likely to engage in greenwashing are the dirtiest ones, because dirty companies know they have a bad reputation, so little is lost in making a green claim if the opportunity arises. At the same time, he and coauthor John W. Maxwell wrote in 2006, “[P]ublic outrage over corporate greenwash is more likely to induce a firm to become more open and transparent if the firm operates in an industry that is likely to have socially or environmentally damaging impacts, and if the firm is relatively well informed about its environmental social impacts.”^19^

“It’s somewhat counterintuitive, but the clean guy is likely to shut up altogether,” Lyon says. “The rationale is: if you’re clean and people already think you’re a green company, you don’t need to bother touting it so much—and if touting it puts you at risk of being attacked, just shut up and let people think you’re clean.”

## Making Green Claims Work

But when a clean company pulls in its horns over the risks of backlash from a cynical public, Lyon believes an opportunity has been lost. He suggests clean companies can be effective green marketers if they take certain steps. First, he says, they might incorporate a full-blown environmental management system (EMS), which would detail its full environmental program in a comprehensive manner. “When a company has an EMS in place, you have a greater expectation that they actually do know what their environmental results are,” Lyon explains. EMSs themselves are supposed to meet an international standard called ISO 14001 developed by the nongovernmental International Organization for Standardization in Geneva, which sets out a variety of voluntary environmental standards.

Another step is to take part in the Global Reporting Initiative (GRI), an international organization that has pioneered the world’s most widely used corporate sustainability reporting framework. The GRI was launched in 1997 by a nonprofit U.S. group called Ceres—a network of investors, environmental organizations and other public interest groups—in partnership with the United Nations Environment Programme. Lyon says the GRI can provide good green credibility at the company level.

The FTC’s attention, however, is directed at products—not companies. And Lyon is one of several experts questioning how effective the looming changes to the Green Guides will be in modifying greenwashing. “Honestly, I don’t think the FTC Green Guides are going to block much activity,” he says. “All the FTC can do is force companies not to provide materially false information. They could potentially go into the domain of what’s misleading as well, but that’s very tricky. But they could . . . require companies to give you a more complete story.”

To Lyon, the ideal system for regulating green marketing claims would entail comprehensive labeling and certification requirements. “You could picture a system that would be a little like the nutrition labeling that we get for food,” he explains. “But whether or not that would be helpful is really unclear to me. From what I understand, there’s not a lot of evidence that those nutrition labels have changed America’s eating habits.”

Among hundreds of green labels available today, a few are broadly recognized as highly reliable. One of them is Green Seal, which awards its seal to companies that meet standards that examine a product’s environmental impact along every step of the production process, including its supply of raw materials. “It’s a differentiator,” says Linda Chipperfield, vice president of marketing and outreach at Green Seal. “If you’re really walking the walk, you should be able to tell your customers about it.”

Other labels are attained via self-certification—that is, if a company wants the label, they can buy it—and aren’t so reliable. The Government Accountability Office (GAO) recently proved that in an investigation^20^ of Energy Star, a joint program of the U.S. Environmental Protection Agency (EPA) and Department of Energy.

Energy Star provides labels to companies who submit data about products and seek the stamp of approval to place on their packages. “Currently, in a majority of categories [Energy Star] is a self-certification by the manufacturer, which leaves it vulnerable to fraud and abuse by unscrupulous companies,” says Jonathan Meyer, an assistant director in the GAO’s Dallas office. Indeed, over a nine-month period, GAO investigators gained Energy Star labels for 15 bogus products, including a gas-powered alarm clock the size of a portable generator. In addition, two of the bogus firms that GAO created as “manufacturers” of the products received phone calls from real companies that wanted to purchase products because the fake companies were listed as Energy Star partners.^20^

The EPA and DOE subsequently issued a joint statement pledging to strengthen the program.^21^ The GAO report has also prompted responses from consumers and industry alike that a strong and reliable federal certification program is needed. In a story on the investigation *The New York Times* quoted the director of customer energy efficiency at Southern California Edison as saying industries affected by Energy Star hope the report will be “a wake-up call to whip [the program] into shape.”^22^

Case believes an improved regulatory scheme does require some kind of certification and labeling. “I think there is room for some kind of unifying green label,” he says. “But I’m not sure if the government wants to get into the business of putting ‘approved’ stickers on good products.” He proposes that the function of providing environmental labels be handled by a new office of the EPA. Under this plan, the EPA would combine several existing environmental labels (such as Energy Star and Green Seal) under a single brand to make it easier for consumers to identify more environmentally preferable goods and services. He points to the U.S. Department of Agriculture’s affirming label on organic foods as a model.

## Toward a Unified Approach

The growing demands of society for greener products and corporate America’s desires to meet it and make a profit make for “a fascinating interaction with cultural change,” says Lyon. “The norms have really started to shift. I think that’s our hope for information and labeling—that it will create a new floor that keeps rising. I don’t think we’re anywhere close to that yet, but I think it’s starting to happen.”

Case says he is “somewhat hopeful” that all involved are moving toward a unified approach to solving the challenges posed by greenwashing. “The huge danger of green-washing is if consumers get so skeptical that they don’t believe any green claims,” he says. “Then we’ve lost an incredibly powerful tool for generating environmental improvements. So we don’t want consumers to get too skeptical.”

## The Seven Sins of Greenwashing

In the course of assessing thousands of products in the United States and Canada, TerraChoice Environmental Marketing categorized marketing claims into the following “seven sins of greenwashing”:

1. **Sin of the hidden trade-off:** committed by suggesting a product is “green” based on an unreasonably narrow set of attributes without attention to other important environmental issues (e.g., paper produced from a sustainably harvested forest may still yield significant energy and pollution costs).
2. **Sin of no proof:** committed by an environmental claim that cannot be substantiated by easily accessible supporting information or by a reliable third-party certification (e.g., paper products that claim various percentages of postconsumer recycled content without providing any evidence).
3. **Sin of vagueness:** committed by every claim that is so poorly defined or broad that its real meaning is likely to be misunderstood by the consumer (e.g., “all-natural”).
4. **Sin of irrelevance:** committed by making an environmental claim that may be truthful but is unimportant or unhelpful for consumers seeking environmentally preferable products (e.g., “CFC-free” is meaningless given that chlorofluorocarbons are already banned by law).
5. **Sin of lesser of two evils:** committed by claims that may be true within the product category, but that risk distracting the consumer from the greater health or environmental impacts of the category as a whole (e.g., organic cigarettes).
6. **Sin of fibbing:** committed by making environmental claims that are simply false (e.g., products falsely claiming to be Energy Star certified).
7. **Sin of false labels:** committed by exploiting consumers’ demand for third-party certification with fake labels or claims of third-party endorsement (e.g., certification-like images with green jargon such as “eco-preferred”).

Adapted from: ***The Seven Sins of Greenwashing: Environmental Claims in Consumer Markets***^2^

## Making Sense of All Those Labels

Although the sheer number of green labels can make it hard to tell legitimate claims from bogus ones, not all ecolabeling is greenwash. Many certifications and labels offer useful guidance for selecting products and services that really are produced in more sustainable fashion. Now there are resources to help consumers judge the labels they encounter.

Visitors to http://ecolabelling.org/ can search more than 300 labels by any of 10 categories (buildings, carbon, electronics, energy, food, forest products, retail goods, textiles, tourism, and other) or by world region. The site explains what products the label is used for and the steps producers and manufacturers must follow to obtain certification. Visitors can also rate and discuss each label.

*Consumer Reports’* Eco-Labels Center at http://www.greenerchoices.org/eco-labels/lets users search for information by label, product category, or certifying organization or program. Each label receives a “report card” and an extensive evaluation describing the certification requirements for the label, the type and extent of input solicited in crafting those requirements, how certification is verified and by whom, and the funding and structure of the certifying body. The evaluations also tell how meaningful the label is for each product type (for instance, the USDA Organic label is deemed highly meaningful for foods but not for cosmetics). The site also describes the elements of a “good” label and offers a glossary of terms used on labels.

## Figures and Tables

**Figure:**
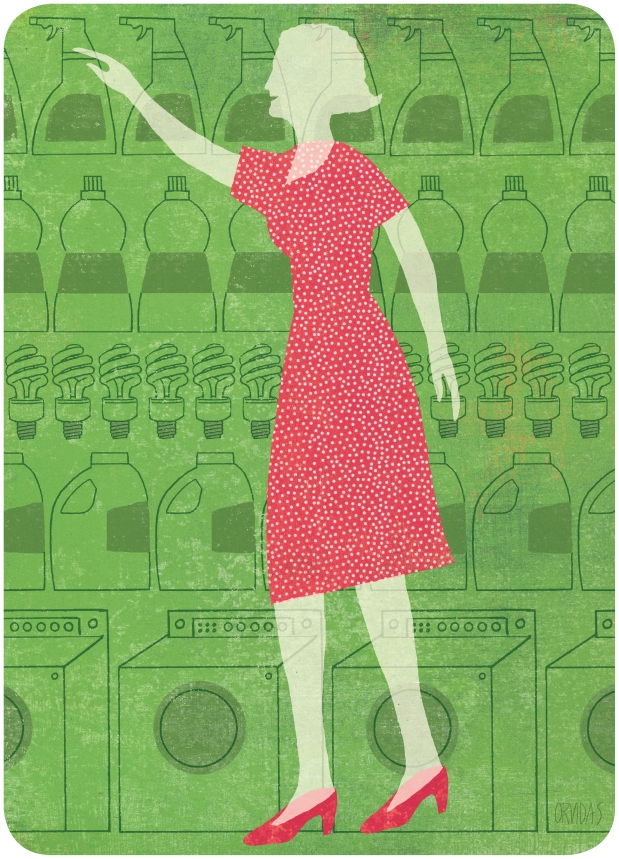


**Figure:**
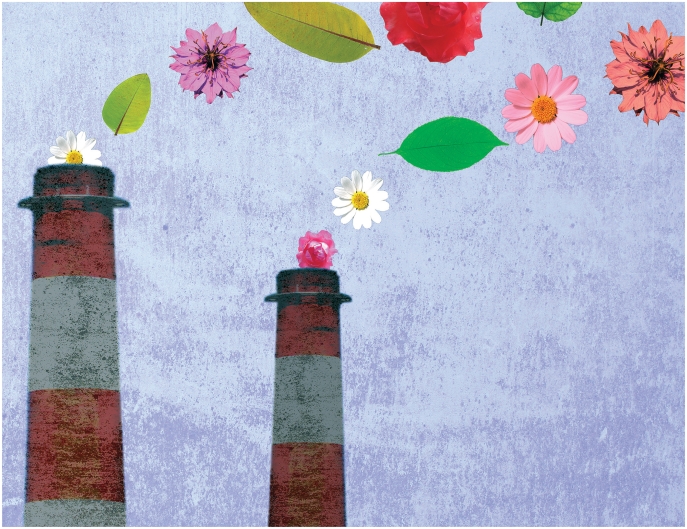


**Figure:**